# Persistent Primitive Olfactory Artery in Serbian Population

**DOI:** 10.1155/2013/903460

**Published:** 2013-07-14

**Authors:** Ljiljana Vasović, Milena Trandafilović, Slobodan Vlajković, Ivan Jovanović, Slađana Ugrenović

**Affiliations:** ^1^Department of Anatomy, Faculty of Medicine, University of Niš, Boulevard Dr. Zoran Đinđić 81, 18000 Niš, Serbia; ^2^Center for Biomedical Researches, Faculty of Medicine, University of Niš, Boulevard Dr. Zoran Đinđić 81, 18000 Niš, Serbia

## Abstract

The continuation of the cranial branch of the primitive internal carotid artery is called the primitive olfactory artery (PO**ℓ**A). It takes this name according to the fact that it is mainly concerned with supplying the developing nasal region. We reported two new cases of the persistent PO**ℓ**A (PPO**ℓ**A) in Serbian population after retrospective analysis of digital images of 200 fetal and 269 adult cases. This PPO**ℓ**A originated from the precommunicating part (A1) of the right anterior cerebral artery, coursed along the olfactory tract, and turned on the medial cerebral hemisphere in both male adults. Some vascular variations (fenestration of the A1 and the median artery of the corpus callosum) were associated with this persistent vessel. According to the fact that we did not find aneurysm in our previous and two recent cases, we are of the opinion that PPO**ℓ**A is usually asymptomatic in Serbian population.

## 1. Introduction

During embryonic development of the vascular system, the primitive olfactory artery (PO*ℓ*A) represents a continuation of the cranial branch of the primitive internal carotid artery (ICA) [1]. It may be presumed that a fenestration of the anterior cerebral artery (ACA) is the remnant of the embryonal plexiform anastomosis between the ACA and the PO*ℓ*A [2, 3]. Some authors noted that PO*ℓ*A normally regresses to the remnant—the recurrent artery of Heubner (RAH) [4, 5]. Other authors supported this claim by the evidence of an aplasia of the RAH [6–8] and/or the anterior communicating artery [6, 9]. 

A persistence of the PO*ℓ*A (PPO*ℓ*A) is very rare according to the fact that its incidence was noted in 0.14% [10] to 0.29% [11] of cases. 

Morphologically, the PPO*ℓ*A usually courses anteromedially along the ipsilateral olfactory tract, and after a turn, it supplies the ACA territory [4]. Pathoanatomically, a relatively frequent location of an aneurysm on the hairpin bend of the PPO*ℓ*A emphasizes the importance of hemodynamic stress in this persistent primitive vessel [7, 12, 13]. 

Reports of single cases or retrospective studies about special and/or general features of the PPO*ℓ*A came usually from Japan [5–10, 12–17] and Korea [11, 18, 19], although there were case reports from UK [20], Taiwan [21], and Serbia [22]. 

Previous incidental finding of the PPO*ℓ*A in one adult cadaver [22], and the description of fenestration of the precommunicating part (A1) of the ACA [3] and/or RAH in fetuses [23], inspired us to a more detailed investigation of the PO*ℓ*A persistence in Serbian population. 

## 2. Materials and Methods

We did a retrospective analysis of digital images of brain vessels of 200 fetal and 269 adult cadavers, dissected at the Department of Anatomy and Institute of Forensic Medicine in Niš, respectively. 

### 2.1. Fetal Cadavers

Fetuses of both genders, from 9 to 32 weeks of gestation, were a part of the collection of our Department of Anatomy, and they were used in the preparation of doctoral thesis [24]. All fetuses were obtained legally from the Clinic of Gynecology and Obstetrics in Niš. The Council for Postgraduate Study of the Faculty of Medicine in Niš gave permission to investigate the fetal material. The arteries of fetuses were injected with Micropaque or latex through the left cardiac ventricle or through the common carotid artery. All fetuses were fixed in 10% formalin for 2 weeks. Fetal brains were removed and kept in individual calvarias. The measurements were performed by means of an ocular micrometer mounted on an operating microscope (Olympus). 

### 2.2. Adult Cadavers

The dissected brains originated from cadavers of both genders, different ages (from the neonate to 95 years), and different causes of death in the period between 2006 and 2013. Investigation of these cases was in accordance with the rules of the internal Ethics Committee (no. 01-9068-4) of our Faculty of Medicine. Morphological features of brain arteries (caliber, possible vessel's abnormalities) were observed by using a magnifying glass and recorded on a film. Measurement of the external diameter of arteries was performed by ImageJ (http://rsb.info.nih.gov/ij/index.html).

## 3. Results and Discussion

### 3.1. Results

We discovered two new cases of the PPO*ℓ*A in adults. The first case of the PPO*ℓ*A was found in a male cadaver, aged 58 and autopsied due to cardiac arrest; another case was found in a male, aged 61, who died due to polytrauma at the Orthopedic Clinic.

**Figure 1:**
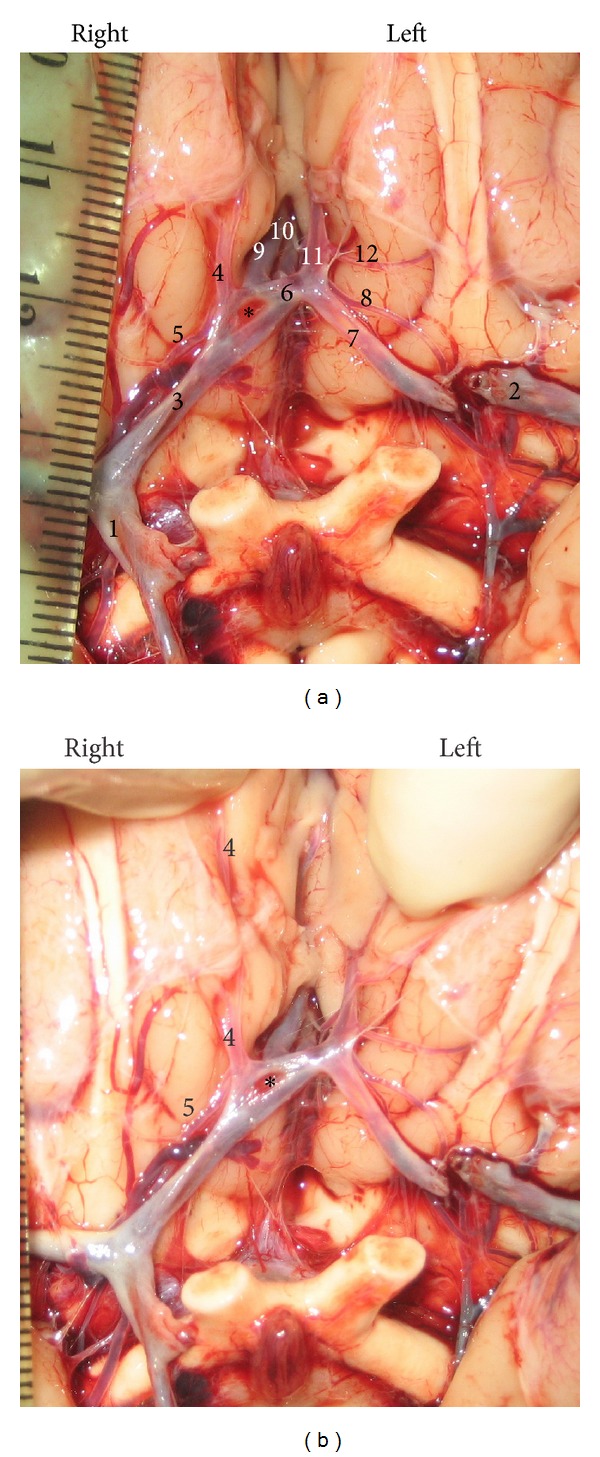
Persistent primitive olfactory artery (PPO*ℓ*A) as a side branch of the precommunicating part (A1) of the right anterior cerebral artery (ACA). It originates at the level of proximal end of the A1 fenestration (a) and courses to the medial surface of the frontal lobe (b). Cerebral part (C4) of the right internal carotid artery (1); left C4 (2); right A1 (3); A1 fenestration (∗); right PPO*ℓ*A (4); right recurrent artery of Heubner (RAH) (5)); anterior communicating artery (ACoA) (6)); left A1 (7); left RAH (8); right postcommunicating part (A2) of the ACA (9); median artery of the corpus callosum (10); left A2 (11); left medial frontoorbital artery (12).

**Figure 2:**
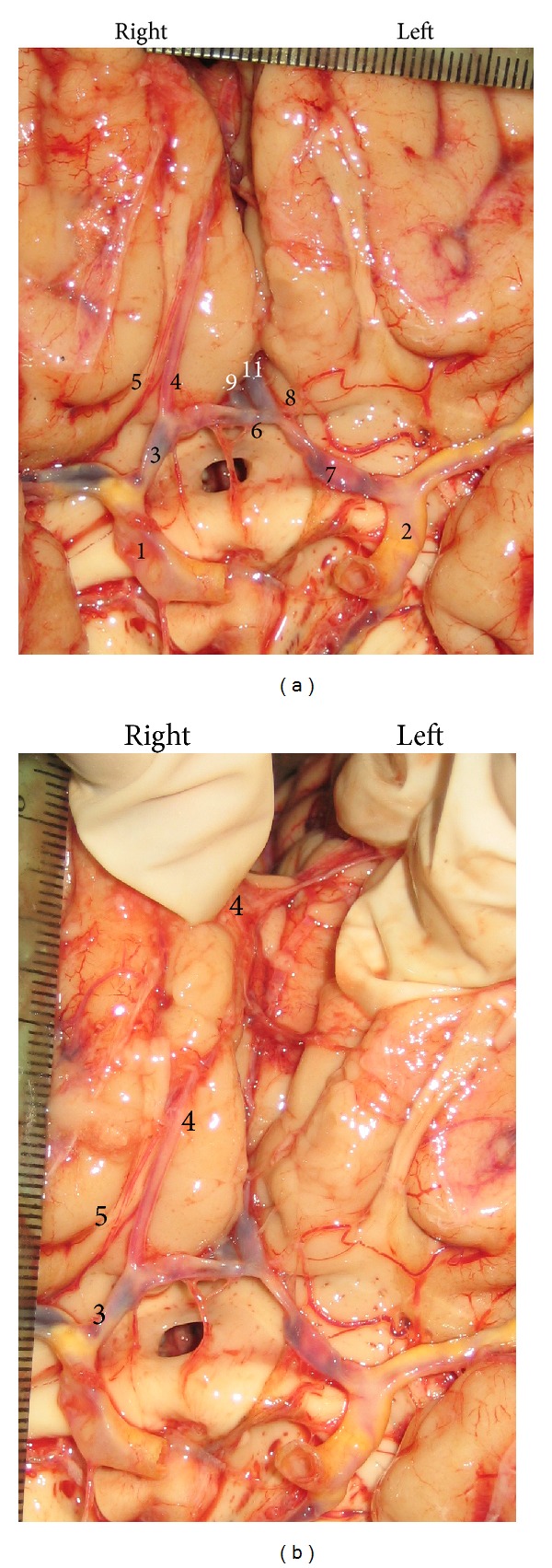
Persistent primitive olfactory artery (PPO*ℓ*A) as a side branch of the precommunicating part (A1) of the right anterior cerebral artery (ACA). It originates from the anterior wall of the A1 (a) and courses to the medial surface of the frontal lobe, where the PPO*ℓ*A gave off a bihemispheric branch (b). Cerebral part (C4) of the right internal carotid artery (1); left C4 (2); right A1 (3); right PPO*ℓ*A (4); right recurrent artery of Heubner (RAH) (5)); anterior communicating artery (ACoA) (6); left A1 (7); left RAH (8); right postcommunicating part (A2) of the ACA (9); left A2 (11).

1. *Case I.* The PPO*ℓ*A had a common origin with the RAH from the A1 of the right ACA at the level of the proximal part of the fenestration (Figure 1(a)). Its beginning was about 7 mm away from ICA bifurcation. The caliber of the right A1 was 2.03 mm, whereas the caliber of the PPO*ℓ*A was 1.41 mm. The latter followed the olfactory tract in the first part of its course, and after that, it turned and passed on the medial telencephalic surface. However, we did not photograph its termination. In addition, the median artery of the corpus callosum was also presented (Figure 1(b)).
2. *Case II.* The PPO*ℓ*A was a branch of the right A1 (Figure 2(a)). The beginning of the PPO*ℓ*A was about 6 mm away from ICA bifurcation. The caliber of the right A1 was 2.50 mm, whereas caliber of the PPO*ℓ*A was 1.40 mm. The latter followed the olfactory tract in the first part of its course, and after that, it passed similar to the callosomarginal artery; a bihemispheric branch was also evidenced at the level of its termination (Figure 2(b)). Ipsilaterally, the RAH originated from the PPO*ℓ*A; it was duplicated at the beginning. Atherosclerotic plaques were significantly present at the cerebral arteries, especially along main brain arteries.
3. *Comparison of* PPO*ℓ*As *in the Literature.* General and special data about PPO*ℓ*As in our and other populations are presented in Table 1.

### 3.2. Discussion

Firstly, we noted some data and disagreements in the literature about the PO*ℓ*A origin [1, 6, 25], PO*ℓ*A rudiments [1] or a lack of embryologic explanation of A1 variable side branches [15, 26], as well as an origin [20] or termination [8, 15] of the PPO*ℓ*A. 

Moffat [1] described that PO*ℓ*A has a similar development in the rat's and human embryos up to an 18 mm stage. During the development of vascular system in a 3.7 mm embryo, the continuation of the dorsal aorta forms the primitive ICA [1], except for its first segment which was formed by the primitive third aortic arch [6]. At the level of the forebrain the ICA gives the primitive maxillary artery, and then ICA divides into the cranial and caudal branches. In embryos of 4 to 5.7 mm (28–30 days), the cranial branch constitutes the primitive olfactory artery (PO*ℓ*A), which branches off the anterior choroidal and middle cerebral arteries [6]. According to data from the paper by Horie et al. [13], the definitive ACA extends superiorly between the cerebral hemispheres, associated with regression of the PO*ℓ*A untill the 7th week of gestation. According to the picture of human embryo in the paper by Katayama et al. [14], the PO*ℓ*A retains its origin from the A1 to the 9 mm embryonic stage. Komiyama [25] and Okahara et al. [6] stressed that the PO*ℓ*A terminates in the nasal fossa, and “secondary artery” constitutes the medial olfactory artery, which supplies the olfactory bulb. This medial olfactory artery becomes the ACA proper in an 11.5 to 18 mm embryo, while the terminal portion of the PO*ℓ*A usually regresses. Kim et al. [27] noted the existence of the plexiform anastomosis between the ACA and the PO*ℓ*A in the illustration of a 14 mm embryo. Lateral olfactory branches of the PO*ℓ*A include the RAH, anterior choroidal, lateral striate, and middle cerebral arteries. Moffat [1] noted that the PO*ℓ*A in a 24 mm human embryo forms an inconstant striate branch of the ACA. 

Okahara et al. [6] noted that the PPO*ℓ*A arises only from the A1 part, as in cases described by Moffat [20] and Horie et al. [13], as well as in recent cases. In addition, we could compare the origin of PPO*ℓ*A from the A1 part in these cases with the PO*ℓ*A origin in an 18 mm human embryo whose picture was displayed in the paper by Katayama et al. [14]. Tsutsumi et al. [8] described that PPO*ℓ*A originated from A1-A2 junction. However, many authors described its internal carotid origin [7, 9, 14, 16, 19, 21]. In our previous case the PPO*ℓ*A and posterior communicating artery had a common origin from the middle cerebral artery (MCA) [22]. It can be said that the case, described by Lin et al. [21], in which PPO*ℓ*A continued from an accessory MCA, resembles our case. Interesting anomaly was found in five rat embryos studied. In these specimens the cranial branch of the ICA terminated as the MCA, whereas the PO*ℓ*A was a continuation of the primitive maxillary artery [1]. 

Several types of the PPO*ℓ*A are described in the literature. In the first type described by Nozaki et al. [7] the PPO*ℓ*A rose from the ICA, ran along the olfactory tract, and made a hairpin bend to supply the territory of the ACA postcommunicating part (A2). In the second type, described by the same authors, the PPO*ℓ*A rose from the ACA and passed through the cribriform plate of the ethmoid bone to supply the nasal cavity, similar to the ethmoidal arteries. The case described by Enomoto et al. [15], on computer tomography angiography (CTA), and the case described by Moffat [20] on autopsy were classified as the second type. Komiyama [25] noted that this second type of the PPO*ℓ*A is homologous to the internal ethmoidal artery in the dog. In the third (transitory) type described by Horie et al. [13], the PPO*ℓ*A divided into two branches; one artery was similar to Nozaki's type 1, whereas the second one had features of Nozaki's type 2. In our “third” type, the PPO*ℓ*A of the MCA origin had a common trunk with posterior communicating artery (PCoA) and coursed forward to the ipsilateral olfactory tract [22]. The case of the PPO*ℓ*A termination (PPO*ℓ*A—cortical frontal vein shunt) described by Tsutsumi et al. [8] was classified as type 4. 

Medial frontoorbital and frontopolar arteries are different from the PPO*ℓ*A according to the beginning (A2 part) and their course. Exceptions were possible as in cases described by Enomoto et al. [15] and Krishnamoorthy et al. [26]. 

We described some cases of the PO*ℓ*A partial persistence in human fetuses [23]. Recently, we described these two cases of the PPO*ℓ*A, found only among adult cadavers. Previous [22] and recent cases indicate that the incidence of the PPO*ℓ*A is 0.64% in Serbian population. These incidences were 0.14% in the Japanese population [10] and 0.29% in the Korean population [11]. For the purpose of calculating of number of published cases, we included some paper abstracts [12, 14–17] in the list of references. Based on the case numbers in our Table 1 and Moffat's allegation about two cases of the PPO*ℓ*A described in 1951 and 1961 by other authors [1], we counted 67 cases to this time. Based also on the cases in the same table, we could note that PPO*ℓ*A was more frequent in males and on the left side, although the PO*ℓ*A persisted on the right side in males of Serbian population, as we noted in our work. The existence of bilateral PPO*ℓ*A was noted five times in the literature [7, 10, 11]. The youngest person was 18 years old [5] and the oldest 82 years [11].

In summary, we did not find any aneurysm on the PPO*ℓ*A in our cases, opposite to some authors who found it on the PPO*ℓ*A hairpin bend [9, 12, 15, 17], and/or on other cerebral arteries [9–11, 13, 14, 16]. Association of the PPO*ℓ*A and other vascular variations were also noted [7, 11, 20, 22], as in our adult cases. 

## 4. Conclusions

The primitive olfactory artery is a rare persistent primitive vessel (0.64%) in Serbian population. It was incidental finding in presented cases. We did not find any complete vascular trunk in fetuses, but we did in adults of male gender, on the right side. 

## Figures and Tables

**Table 1:** Comparison of general and special data about the persistent primitive olfactory artery (PPO*ℓ*A) presented in our and other investigations.

Country [authors]	Age*	Gender (no.)	Initial symptoms	Diagnosis	PPO*ℓ*A							
					Incidental finding	Relation of number cases	Vascular source	Side	Nozaki's and new types**	Associated variations (no.)	Aneurysmatic artery (no.)	Other cerebral pathology
*Single cases *												
Korea [18]	24	F	Vertigo	3D CTA	+		ICA	R	I			
Serbia [22]	35	M	Myoc. infarction	Autopsy	+		ICA^p^	R	III	Origin/end of the right PCoA		
Japan [14]	42	M		3D CTA	+		ICA	L	I		ACoA	
Japan*** [15]	44	M	Headache	CTA	+				(II)	Anastom. with left ant. ethm. a.	Left fronto-orbital artery (PPO*ℓ*A)	Intracerebral hem./SAH
Japan [7]	54	F	Anosmia	Cereb. angio.	+		ICA	L/R	I	ACoA apl. RAHs apl.		
Japan [14]	55	F		Cereb. angio.	+		ICA	L	II		ICA	
Japan*** [16]	59	F			+		ICA	L	I		ACA	
Japan*** [17]	59	F	Hyposmia		+		ICA	R			PPO*ℓ*A	
Japan [8]	59	M	General seizure	CTA	+		A1-A2	R	(IV)	PPO*ℓ*A- cort. front. vein shunt RAH apl.		Intracerebral hem.
Taiwan*** [21]	62	F		Cereb. angio.	+		ICA	R	(I)	Moyamoya ph. Fetal PCA MCA acc.		MCA occl.
Korea [19]	68	F	Headache	CTA	+		ICA	R	(I)	PPO*ℓ*A bulbous dilatation		
Japan [9]	69	M	Loss of consciousness	3D CTA	+		ICA	R	(I)	ACoA apl.	PPO*ℓ*A/left A1-A2/ ICA	
UK [20]	71	M	Gastric carcinoma	Autopsy	+		A1	L	(II)	Double ACoA		
Japan [13]	78	M	Headache	Axial DynaCT/DSA	+		A1	R	Transitory type (I/II)	PPO*ℓ*A- ethmoidal anastomosis	Right A1	SAH
*Retrospective studies *												
Japan [5]		M (1)	*Clinical or forensic * *investigations *	MRA	1/900		ICA	R				
Japan [6]				CTA/MRA	1/3700		ICA	L				
Japan*** [12]					5/?		ICA				PPO*ℓ*A (1)	
Korea [11]	24 −82	M (17) F (12)		MRA/CTA	29/9841			L (19) R (7) LR (3)		ACA hypop. (3) M1 fen (1)	PPO*ℓ*A (2) ICA (1) Three cereb. arteriæ (1)	
Japan [10]	36 −81	M (6) F (8)		MRA	14/3491			L (7) R (6) LR (1)		VA fen. (1) MCA acc. (1) MACC (1)	MCA (1) PPO*ℓ*A (1)	
Serbia (*recent study*)	0 −95	M (2)		Autopsy	2/469		A1	R (2)	I	A1 fen (1) MACC (1)		

*Single cases are aligned according to the age.

**Nozaki's [7] and new types are marked by Romanian numbers.

***Data from the paper by Katayama et al. [14].

Female (F); male (M); left (L); right (R); myocardial (myoc.); three-dimensional computer tomography angiography (3D CTA); cerebral angiography (cereb. angio.); digital subtraction angiography (DSA); magnetic resonance angiography (MRA); internal carotid artery (ICA); persistent cranial branch of the internal carotid artery (ICA^p^); junction of the precommunicating and postcommunicating parts (A1-A2) of the anterior cerebral artery (ACA); posterior communicating artery (PCoA); aplasia of the anterior communicating artery (ACoA apl.); recurrent artery of Heubner (RAH); accessory middle cerebral artery (MCA acc.); posterior cerebral artery (PCA); fenestration of the sphenoid part of the MCA (M1 fen); vertebral artery (VA); median artery of the corpus callosum (MACC); hemorrhage (hem.); occlusion (occl.); subarachnoid hemorrhage (SAH).
